# On the proper reading of the TBRS model: reply to Oberauer and Lewandowsky (2014)

**DOI:** 10.3389/fpsyg.2014.01331

**Published:** 2014-11-21

**Authors:** Pierre Barrouillet, Valérie Camos

**Affiliations:** ^1^Developmental Cognitive Psychology, Faculté de Psychologie et des Sciences l'Education, Université de GenèveGenève, Switzerland; ^2^Laboratory of Cognitive Development, Département de Psychologie, Fribourg Center for Cognition, Université de FribourgFribourg, Switzerland

**Keywords:** working memory, complex span, decay, cognitive load, TBRS model

The limitations of working memory (WM) and their determinants have been a matter of intense debate in the last years (e.g., Barrouillet and Camos, 2012; Oberauer and Lewandowsky, 2013; Ricker et al., 2014; see Barrouillet and Camos, 2015, for a review). Is forgetting from WM only due to interference, or do WM traces also suffer from temporal decay? This latter hypothesis has recently gained some credence from the verification of the main predictions of the time-based resource-sharing model (TBRS, Barrouillet et al., 2004), a theory that describes WM functioning as the interplay between the antagonistic processes of temporal decay and restoration of memory traces. However, Oberauer and Lewandowsky (2014) have lately claimed that the predictions that the authors of the TBRS model drew from their theory do not follow from its main tenets. In this rejoinder, we show that Oberauer and Lewandowsky's analysis is wrong, revealing a misunderstanding of the TBRS model.

## The TBRS model and its predictions

**Figure 1:**
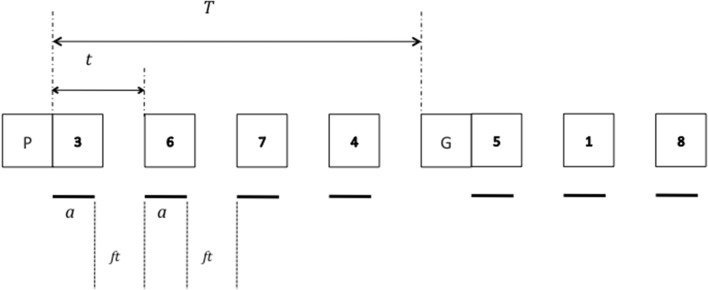
**Illustration of the temporal parameters relevant for recall performance in a complex span task according to the TBRS model**.

The TBRS model was initially developed to account for performance in complex span tasks involving the maintenance of memory items (e.g., words or digits) while performing an intervening activity (e.g., solving equations). Assuming that memory traces decay when attention is switched away, but are refreshed when attention is available anew, we predicted that WM spans (i.e., the maximum number of items that can be recalled) should be a function of the proportion of time during which the intervening activity occupies attention. We called this proportion cognitive load (CL, Barrouillet et al., 2004). To test this hypothesis, we designed new computer-paced WM tasks in which participants are presented with series of memory items for further recall (e.g., letters), each of them being followed by a series of distracters successively displayed on screen at a fixed pace (e.g., digits for a parity judgment task). Figure 1 illustrates such a task, black bars below each digit representing the duration of the attentional capture involved by parity judgment. Let us call *a* this duration, *N* the number of distracters to be processed after each memory item, and *T* the total time available to process them (i.e., the time elapsed between two successive memory items). Cognitive load would thus correspond to

(1) CL=aN/T

We verified in several studies that WM spans are a linear function of CL with lower CL resulting in higher spans (Barrouillet et al., 2004, 2007, 2011a; Vergauwe et al., 2010; see Barrouillet and Camos, 2012, 2015, for reviews). It is worth noting that *CL* depends on the pace at which distracters are processed, but not on their number. This appears when expressing *T* in Equation (1) as *tN*, with *t* referring to the time allowed to process each distracter (Figure 1).

(2) CL=aN/tN=a/t

It becomes clear that *N* can vary without affecting *CL*. This is why the TBRS model predicts that, provided that the distracters are presented at a constant pace, their number has no effect on WM performance because *CL* remains unchanged. This prediction was verified in several studies (Barrouillet et al., 2004, 2011b; Plancher and Barrouillet, 2013). However, Oberauer and Lewandowsky (2014) have recently claimed that the TBRS model does not warrant such a prediction, and that the absence of effect of the number of distracters rules our theory out.

## Oberauer and Lewandowsky's (2014) analysis of the TBRS model

Oberauer and Lewandowsky assume that, within the TBRS model, each distracter operation *i* involves a certain loss of memory strength Δ*di* by decay due to attentional capture. This capture is followed by a period of free time *fti* during which memory traces can be refreshed resulting in a gain in memory strength Δ*ri*. They conclude that the net loss from processing a distracter *i* is therefore Δ*di* - Δ*ri*. If Δ*D* is the mean loss of memory strength through decay across all distracter operations in the interval between two memory items, and Δ*R* is the mean gain of memory strength through refreshing, the net loss after *N* distracters should equal *N(*Δ*D* - Δ*R)*. Thus, Oberauer and Lewandowsky assume “if, at a given level of CL, Δ*D* exceeds Δ*R*, then the net loss of memory strength necessarily increases with *N*, which inevitably leads to the prediction of more forgetting with increasing number of distracter operations at a constant level of CL. Thus, memory must be predicted to depend not only on CL but also on a second variable, *N*” (Oberauer and Lewandowsky, 2014, p. 17). Oberauer and Lewandowsky envision only one exception to this conclusion. This is when Δ*R* fully compensates Δ*D*, in which case processing distracters would no longer impair memory at all compared to a no-distracter control, something that could only happen, in their view, for very low levels of CL. However, for any higher level of CL, they consider that Δ*D* - Δ*R* must be larger than 0, implying that memory performance must be predicted to decline with increasing *N*. In other words, Oberauer and Lewandowsky claim that the TBRS model predicts an effect of the number of distracters, even at constant CL. They consider their analysis as buttressed by the results of computational simulations run with their TBRS^*^ model (Oberauer and Lewandowsky, 2011) that they present as a computational implementation of the TBRS model. TBRS^*^ produced more forgetting with a greater number of distracters, except for very low CL levels.

Oberauer and Lewandowsky (2014) ran two experiments using the same design as we used in Barrouillet et al. (2011b), a study in which we verified that the number of distracters has no effect on recall performance at constant CL, and they replicated our findings. Based on their own analysis of the TBRS model, they claimed that the absence of effect of the number of distracters at constant CL would contradict it.

## A reappraisal of Oberauer and Lewandowsky's (2004) analysis

Although Oberauer and Lewandowsky's analysis seems to reflect TBRS proposals, it actually neglects the precise mechanisms on which TBRS predictions are based. Let us recall that these predictions concern WM spans, that is the maximum number of memory traces that can be maintained in a state appropriate for their retrieval while performing a given concurrent task. The TBRS model predicts that this number should remain constant across variations of the number of distracting episodes provided that CL remains constant. The critical point here lies in the functional differences between temporal decay and refreshing. Whereas temporal decay is a passive and parallel process that affects all the stored memory traces at the same time, refreshing is an active and serial process directed to one memory trace at a time (McCabe, 2008; Barrouillet et al., 2011a). Thus, whereas memory decay does not depend on the number of memory traces stored in WM, refreshing does. To anticipate, a given free time *ft* can be sufficient for refreshing *n* memory items in such a way that the Δ*R* fully compensates the Δ*D* of each of these memory traces, but not sufficient for refreshing *n + 1* items, resulting in a failure to recall the entire list and in a WM span limited to *n*.

Let us call *Si* the strength of a given memory trace that determines its accessibility for refreshment or recall. Let us also assume that there exists some minimum strength level *Sm* below which a memory trace cannot be retrieved for refreshing or recall and is consequently lost. For sake of simplicity, suppose that, at the beginning of a processing episode involving a decrement Δ*D*, all the memory traces stored in WM have the same strength *S*. Their strength *Se* at the end of this episode will be:

(3) Se=S−ΔD

If these memory traces can be retrieved after this processing episode, that is if *Se ≥ Sm*, WM span will be determined by the maximum number of items that can benefit for a refreshing Δ*ri* that is sufficient to permit them to survive the next processing episodes and their associated decrement Δ*D*. This means that the value Δ*ri* for a given memory item must satisfy the inequality

(4) Se+Δri−ΔD≥Sm

Basically, considering that *Se* ≥ *Sm* (if this is not the case, the item can not be retrieved and refreshed), the inequality will be verified each time Δ*ri* equals or exceeds Δ*D*. However, this condition is not necessarily satisfied for all the memory items to be maintained. If *tr* is the time needed by the refreshing mechanism to produce the requested Δ*ri* on a single memory trace, and *ft* the free time available for refreshing memory traces before the next distracting episode, the maximum number of items that can be sufficiently refreshed to survive this next processing episode is the integer *n* such as

(5) n=[ft/tr]

Importantly, none of the parameters determining *n* depends on the number of processing episodes. The time *tr* needed to refresh a memory trace depends on its strength level *Se* after the processing episode (weaker traces necessitating longer refreshing times), *Se* depending in turn on the duration of the attentional capture *a* involved by this processing episode (longer attentional capture resulting in stronger decay and lower *Se* values). The other parameter in Equation (5), *ft*, represents the free time after a processing episode (see Figure 1). Thus, *n* depends only on the two temporal parameters of the task, *a* and *ft*, which are the sole determinants of CL as expressed by Equation (2), knowing that *t = a + ft*. This can be easily understood in the context of a complex span task in which memory items are presented successively, the length of the lists to be memorized being increased until failure to recall. As the number of items to be maintained progressively increases, there comes a point where it becomes impossible to sufficiently refresh all the memory items in such a way that Δ*ri* compensates Δ*D* for each of them. However, if the free time *ft* allows to compensate the decay Δ*D* of *n* items, this equilibrium can not be modified by adding other distracter episodes involving the same balance between *a* and *ft*.

This is of course true whatever the level of CL, something that Oberauer and Lewandowsky (2014) overlooked. They write, “when an experiment contains multiple CL levels, and performance is found to decline with increasing load, then the equality Δ*R* = Δ*D* that is required for the number of operations to have no effect can only hold for the lowest CL, if at all. At the larger CL levels, Δ*R* < Δ*D* must be true, because otherwise the CL manipulation would have no effect” (Oberauer and Lewandowsky, 2014, p. 19). In other words, Lewandowsky and Oberauer think that a low CL is needed to have no effect of the number of distracters, this effect necessarily appearing at higher CL levels that Lewandowsky and Oberauer attribute to an increased difference between Δ*R* and Δ*D*. This reasoning reveals a misunderstanding of what CL is and what are the determinants of its effect. Indeed, the CL effect is not due to the fact that higher levels of CL correspond to a greater difference between Δ*R* and Δ*D*. Thinking about CL variations as resulting from variations in this difference is misleading because, whatever the CL level, Δ*ri* necessarily equals Δ*D* for each of the recalled items, otherwise they would be lost and forgotten (see Equation 4). As we explained above, CL effects result from the fact that the number of memory items that can be sufficiently restored after processing diminishes with the ratio between free time *ft* and the duration *a* of the preceding attentional capture. For evident reasons, this ratio can not be modified by lengthening the series of distracters when they are presented at an unchanged pace. This is why the TBRS model predicts the absence of effect of the number of distracters at constant CL.

### Conflict of interest statement

The authors declare that the research was conducted in the absence of any commercial or financial relationships that could be construed as a potential conflict of interest.

## References

[B1] Barrouillet P. Bernardin S. Camos V. (2004). Time constraints and resource-sharing in adults' working memory spans. J. Exp. Psychol. Gen. 133, 83–100. 10.1037/0096-3445.133.1.83 14979753

[B2] Barrouillet P. Bernardin S. Portrat S. Vergauwe E. Camos V. (2007). Time and cognitive load in working memory. J. Exp. Psychol. Learn. 33, 570–585. 10.1037/0278-7393.33.3.570 17470006

[B3] Barrouillet P. Camos V. (2012). As time goes by: temporal constraints in working memory. Curr. Dir. Psychol. Sci. 21, 413–419 10.1177/0963721412459513

[B4] Barrouillet P. Camos V. (2015). Working Memory: Loss and Reconstruction. Hove: Psychology Press.

[B5] Barrouillet P. Portrat S. Camos V. (2011a). On the law relating processing and storage in working memory. Psychol. Rev. 118, 175–192. 10.1037/a0022324 21480738

[B6] Barrouillet P. Portrat S. Vergauwe E. Diependaele K. Camos V. (2011b). Further evidence for temporal decay in working memory. J. Exp. Psychol. Learn. 37, 1302–1317. 10.1037/a0022933 21895395

[B7] McCabe D. P. (2008). The role of covert retrieval in working memory span tasks: evidence from delayed recall tests. J. Mem. Lang. 58, 480–494. 10.1016/j.jml.2007.04.004 19633737 PMC2715014

[B8] Oberauer K. Lewandowsky S. (2011). Modeling working memory: a computational implementation of the time-based resource-sharing theory. Psychon. Bull. Rev. 18, 10–45. 10.3758/s13423-010-0020-6 21327362

[B9] Oberauer K. Lewandowsky S. (2013). Evidence against decay in verbal working memory. J. Exp. Psychol. Gen. 142, 380–411. 10.1037/a0029588 22866686

[B10] Oberauer K. Lewandowsky S. (2014). Further evidence against decay in working memory. J. Mem. Lang. 73, 15–30 10.1016/j.jml.2014.02.003

[B12] Plancher G. Barrouillet P. (2013). Forgetting from working memory: does novelty encoding matter? J. Exp. Psychol. Learn. 39, 110–125. 10.1037/a0028475 22563635

[B13] Ricker T. J. Vergauwe E. Cowan N. (2014). Decay theory of immediate memory: From Brown (1958) to today (2014). Q. J. Exp. Psychol. [Epub ahead of print]. 10.1080/17470218.2014.914546 24853316 PMC4241183

[B14] Vergauwe E. Barrouillet P. Camos V. (2010). Verbal and Visuo-spatial working memory: a case for domain-general time-based resource sharing. Psychol. Sci. 21, 384–390 10.1177/0956797610361340 20424075

